# A Prospective, Double-Blind, Randomized, Controlled Clinical Trial in the Gingivitis Prevention with an Oligomeric Proanthocyanidin Nutritional Supplement

**DOI:** 10.1155/2017/7460780

**Published:** 2017-12-10

**Authors:** R. M. Díaz Sánchez, G. Castillo-Dalí, A. Fernández-Olavarría, R. Mosquera-Pérez, J. M. Delgado-Muñoz, J. L. Gutiérrez-Pérez, D. Torres-Lagares

**Affiliations:** Oral Surgery Department, Dental School, University of Seville, Seville, Spain

## Abstract

**Aim:**

To evaluate the effectiveness on tissue response of the new nutritional supplement made of oligomeric proanthocyanidins in induced gingivitis after 21 days of use.

**Material and Methods:**

A prospective, double-blind, randomized, controlled clinical trial was carried out on 20 patients; it is divided into an experimental group and a control group after fulfilling the selection criteria. Patients had to come 4 times during the study to register the Silness and Löe index, the gingival bleeding index, the plaque index, the inflammatory crevicular fluid study (IL6), and the changes in the brightness of the gingiva. No complementary hygiene methods were allowed during the 21 days.

**Results:**

The Silness and Löe index was higher in the control group than in the experimental group, reaching a twofold difference between the groups (*p* < 0.0001). The gingival bleeding index also supports this fact, since the bleeding was lower in the experimental group (*p* < 0.005). However, the dental plaque on the tooth surface according to the plaque index was 33% higher in the experimental group (*p* < 0.006). Some differences in the IL-6 were found in the crevicular fluid (*p* < 0.0001).

**Conclusion:**

Oligomeric proanthocyanidins have an effect on the periodontal tissue's health. No effects on the accumulation of plaque on the tooth surface were found, so further studies are needed to determine the nature of the plaque.

## 1. Introduction

Gingivitis is an inflammatory disease of dental tissue support that in the first instance affects the gingiva. Gingivitis could move on to more advanced stages, leading to the development of periodontitis, in which the inflammation and bacterial infection can produce the destruction of the supporting tissues of the teeth, gingiva, periodontal ligament, and alveolar bone [1–5].

Gingivitis is due to the long-term effects of plaque deposits on the teeth, which is composed of bacteria, located on the surfaces of the teeth and in the gingival sulcus. If plaque is not prevented or removed, it turns into a hard deposit called tartar (or calculus) that becomes trapped at the base of the tooth. Plaque and calculus irritate and produce gingival swelling. Bacteria and the toxins produce infection and more swelling gingiva [1–5].

Crevicular fluid is normal plasma exuded flowing through the gingival sulcus. A patient with gingivitis develops an inflammatory response with a large number of mediators involved, such as the cytokines IL-6 and IL-8 [1–6].

Interleukin 6 (IL-6) is a multifunctional cytokine that is produced by a range of cells, and it is involved in the B-cell differentiation, proliferation, and differentiation of T cells; in the immunoglobulin, stimulation by the B-cell secretion [7] can also induce bone reabsorption [8]. IL6 is a useful diagnostic indicator to determine the progression of the gingivitis to a periodontal disease [9].

The treatment goal of gingivitis is to reduce inflammation by cleaning the teeth, using different instruments to remove the dental plaque deposits according to the case [6]. It is important to educate the patient and prescribe special toothpaste, mouthwash, gel, and so forth which contain a wide spectrum of antiseptic and anti-inflammatory properties. However, the dentist has the responsibility to explain the correct tooth brushing technique to achieve good results [2, 3].

The oligomeric proanthocyanidins (OPCs) are one of the most abundant polyphenolic substances in the plant kingdom. These substances exhibit a range of rather surprising physical and chemical properties which, once applied to living organisms, are translated into a multitude of biological activities [10]. The OPCs have been recently investigated, and, apart from its powerful antioxidant activity, they have been shown to have anticancer, anti-inflammatory, antimicrobial, and vasodilatory properties, revealing to be a potentially valuable therapeutic tool for the treatment of many illnesses [11–15].

The available research has demonstrated that the acidic environment of the human stomach does not easily degrade the proanthocyanidins; therefore, the rates of absorption in the upper gastrointestinal tract are not high [16].

However, it seems that even the low levels observed in urine after an oral dose (generally less than 250/0 of doses/original) are sufficient to significantly increase the antioxidant capacity in plasma/serum. When OPCs reach the colon, they suffer an extensive degradation due to colonic flora [16].

The metabolites and biological properties of this process have not been investigated yet; it has been suggested that they may have protective effects on the antioxidant and systemic illness [11–16].

Regarding the other component included in the OPCs, vitamin C, we know that it is a good associative option to bilberry. Vitamin C generally provides properties that contribute to the formation of collagen, which helps to maintain functionality of teeth and gums and other properties such as reinforcement of the immune system and potent antioxidant opposition to oxidative stress [17–21].

Although in vitro studies published about the interaction between the OPCs and pathogens in the oral cavity have had very good results, there are no publications with the appropriate design to assess the clinical efficacy of OPCs in gingival disease in human, except an article published in 2015 comparing the chlorhexidine and the cranberry mouthwash [22–25].

The aim of this study is to evaluate the effectiveness of the new nutritional supplement made of oligomeric proanthocyanidins (OPCs) on tissue response to prevent gingivitis. We hypothesise a healthy state of the periodontal tissue in the study group versus gingivitis in the control group.

## 2. Material and Methods

### 2.1. Study Design and Population

A randomized, double-blind, placebo-controlled, clinical trial of 21 days of duration was conducted among 20 volunteer students of the School of Dentistry of the University of Seville. The duration of 21 days was chosen as that time allows the parameter study on the gingiva and was enough to study the inflammation of the gingiva without causing any irreversible problem in it. No previous studies have been found in the literature, so 20 subjects were determined for the pilot study to test our hypothesis.

The Ethics Committee of the University of Seville approved the study protocol. Prior to participation, the purpose and procedures were fully explained to all healthy volunteers and all participants gave written informed consent in accordance with the Declaration of Helsinki. The study was designed, conducted, analyzed, and reported according to the guidelines for Good Clinical Practice. The study was approved by the http://ClinicalTrials.gov Protocol Registration and Results System with the number NCT02515929. The protocol can be accessed if necessary in http://ClinicalTrials.gov.

The study was carried out between September 2013 and January 2014. The recruitment started in September, and the baseline stage took place in October 2013. After the 21-day follow-up data was treated obtaining the results in January 2014.

The medical and dental histories were done at the prescreening visit, and we selected the 21 participants based on the inclusion and exclusion criteria. The inclusion criteria were the following: subjects older than 18 years old, male or female, good overall health, and a minimum of 20 teeth (teeth with big caries were crowned or extensively restored, and teeth that were orthodontic banded, abutments, or third molars were not included in the tooth count); the volunteers signed the written consent before the initiation of the study.

The exclusion criteria were the following: periodontal disease (defined as 4 mm and/or positive bleeding when probing), pregnant or breastfeeding, subjects with fixed or removable prosthesis, tumour pathology in oral cavity, use of antibiotics during a 2-month period prior to the start of the trial, hypersensitivity to red fruits in general, xerostomy, active smoker, contagious-infectious pathologies, pathology with severe systemic repercussions, any other judgment that makes the investigator believe to endanger or risk the subject participant, subjects with phenylketonuria or allergy to aspartame, and the use of any oral hygiene product during the study.

A single examiner previously calibrated determined assessment of patient eligibility for the study and enrolment of patients into trial. Patients eligible for the study were individually randomly assigned to oligomeric proanthocyanidins nutritional supplement treatment or placebo groups by an informatics programme by LACER S.A.

The study was double-blind. The examiner and the patient did not know into which group they were assigned. The results were treated by another examiner who knew which patients belonged to the experimental or placebo group. The masking process was established by a number assignation to each patient in the study, and the treatment was the same both for the patient and the examiner who were not allowed to know to which group they belonged.

Patients had to take the experimental or placebo treatment each night after dinner and after a rinse with water. The pill was maintained in the mouth until complete dissolution. Neither drinking nor eating was allowed during 30 minutes after taking the treatment. The experimental treatment consisted of 90 mg exocian cran 408 (equivalent to 36 mg OPCs) and 120 mg of vitamin C, while the placebo group was composed of the same organoleptic substance but free of active ingredients.

Both were similar in appearance. No complementary hygiene methods were allowed during the 21 days (tooth brushing, rinses or irrigation with any product, or flossing), which could constitute a bias for the study.

At the trial baseline stage, a tartar removal was carried out in each patient to regularize the initial situation; a patient diary, enough medication for the entire study, and instructions were given for its correct fulfillment. The inflammation of the crevicular fluid (IL6) and the brightness of the gingiva were registered.

Two evaluation visits were performed on days 14 and 21 of the study for an oral clinical examination and to register the Silness and Löe index, the gingival bleeding index, the Turesky plaque index, the inflammatory crevicular fluid study (IL6), and changes in the brightness of the gingiva.

### 2.2. Silness and Löe Index [26, 27]

Six teeth were examined according to Ramfjord criteria (16-21-24-36-41-44). Four surfaces of each tooth were examined, making a total of 24 measurements. These measurements were performed with a periodontal probe by the same examiner.

### 2.3. Gingival Bleeding Index [28]

A periodontal probe was used to take this index. The values established for this examination are 0—absence of inflammation; 1—mild inflammation, slight change in color, no gingival edema, and no bleeding on probing; 2—moderate inflammation, redness, edema, and gingival hypertrophy, and bleeds to probe (after 10 seconds); 3—severe inflammation, marked redness, and hypertrophy. There may be ulcerations; the gingiva tends to be spontaneous in bleeding.

### 2.4. Turesky Plaque Index [29, 30]

The buccal surfaces of the anterior teeth were examined using a mouthwash of basic fuchsine as developing agent plaque, and the following numerical scoring system from 0 to 5 was established: 0—there is no plaque; 1—independent streaks of plaque in the cervical margin of the tooth; 2—a thin continuous band of plaque (up to 1 mm) at the cervical margin; 3—a band greater than one millimeter wide, but covers less than one-third of the crown; 4—the plaque covers a third, but not more than two-thirds of the crown; 5—the plaque covers two-thirds or more of the crown.

### 2.5. Inflammatory Crevicular Fluid Study (IL6) [7–9]

Crevicular fluid samples were collected from interdental areas (lingual, buccal, mesial, and distal) of six teeth distributed in the four quadrants (16, 21, 24, 36, 41, and 44) using five strips of pressed paper that were 2 cm long, especially for crevicular fluid. The impregnation time for each patient was 5 seconds, and the strips were immediately inserted into microtubes that contained 0.5 ml of Eppendorf with 50 *μ*l saline at 4°C for preservation. The samples were then transported to the biological laboratory in a refrigerator and were stored frozen at −80°C at the laboratory until they were processed.

Then, we proceeded to analyze the concentration (pg/ml) of interleukin 6 present in each sample using panels 96-well bioplex brand Luminex®.

### 2.6. Brightness of the Gingiva

The brightness of the gingiva was taken to identify possible changes in the gingival color. The reddening of the gingiva accompanies the inflammation of the tissue, which is a factor that may help to differentiate inflammatory changes at this level.

The luminosity of the gingiva was registered with Micro SpectroShade™ MHT Optic Research AG. This instrument is designed to take dental color. However, besides including color guides, the SpectroShade has the ability to measure the brightness of any color, not just shades of white. The change of color of the gingiva was observed in each patient, and the color reference was measured in the same point in each evaluation visit, which helped us compare the change in the coloration of the same point.

### 2.7. Statistical Analysis

The management of the data was performed using the SPSS statistical program. A similar database to Notebook Data Collection was created. There are a minimum range and a maximum range for each variable, and the name of the variable and its values were defined. The data are presented as the mean ± standard deviation of the measurements, and a *p* value less than 0.05 was considered statistically significant.

Chi-square test was performed to qualitative variable and Student's *t*-test to the quantitative variable, after the Kolmogorov-Smirnov to assure the normality.

The Ethical Committee approved this study, and all examinations and treatments were performed with the consent of the subjects and according to the guidelines of the Declaration of Helsinki.

## 3. Results

A statistical analysis between the two study groups, 10 patients in each group, in terms of sex, age (years), weight (kg), height (cm), tobacco use, alcohol, and use of concomitant medication, was carried out to ensure the reliability of the results. We found no significant differences between the study group and the control group, so we can say that the homogeneity between the groups was achieved (Table 1).

The medication perception of both groups, as the initial flavour, the product durability, the appearance, size, and efficiency, was the same; there were no statistically significant differences between the data obtained (Table 1). No adverse events were notified during the study.

Once the study groups were analysed, the data processing was conducted depending on the different views held.

### 3.1. Periodontal Index to 15 Days for Treatment (Intermediate Visit)

The Silness and Löe gingival index (of 0–3 from least to greatest gingival inflammation) (Table 2) was higher in the control group than in the experimental group, reaching a twofold difference between the two groups (*p* < 0.0001). The gingival bleeding index also corroborates this fact, as the bleeding was lower in the experimental group versus the control group (*p* < 0.005) (Table 2).

In contrast to the values obtained in the Silness and Löe and gingival bleeding indexes, the amount of dental plaque deposited on the surface of the patients according to the Turesky index plaque was slightly higher (33%) in the experimental group versus in the control group (*p* < 0.006) (Table 2).

### 3.2. Periodontal Index to 21 Days for Treatment (Final Visit)

The results obtained 21 days after starting the treatment showed the tendency observed in the intermediate visit, increasing the difference between the study groups.

Silness and Löe index was higher in the control group compared to the experimental group, with a difference of 50% between the study groups (*p* < 0.0001). The gingival bleeding index, which showed bleeding in the control group, was higher in the experimental group (*p* < 0.0001).

However, the plaque deposition was higher in the experimental group than in the control group according to the Turesky plaque index having plaque deposit doubled in the patients in the experimental group (*p* < 0.0001) (Table 2).

### 3.3. Gingival Brightness

The gingival brightness was taken in each patient using the Micro SpectroShade Optic Research MHT AG. This value was taken at the beginning, middle visit, and in the final visit. The data obtained from these three measurements in each patient showed no statistically significant differences (*p* values from 0.351 to 0.545) (Table 3).

### 3.4. Inflammatory Crevicular Fluid Study (IL6)

Statistically significant differences were found at the baseline between the experimental group and the control group and in the subsequent visits. No statistically significant differences between the first visit and the second visit, and the first and third visits were found, although it was significant between the second visit and the third visit (Table 4).

## 4. Discussion

Gingivitis is caused by deposition of plaque on the tooth surface; this deposition constitutes an irritant that triggers the gingival inflammation and bleeding. Gingivitis could be reversible if the patient has a constant and good hygiene. These measures could be carried out by a proper brushing technique [1–5].

The drug used in the current study is a nutritional supplement based on blueberry and red fruit rich in oligomeric proanthocyanidins (OPCs) [7]. Proanthocyanidins have been recently investigated, as we suggested in the introduction, due to their powerful antioxidant and anticancer activities and due to their anti-inflammatory, antimicrobial, and vasodilator systemical properties. The beneficial effect of proanthocyanidins is attributed to its ability to reduce oxidative stress, lipid peroxidation, free radical generation, and LDL oxidation [11].

Mohana et al. published a study in 2015 about the effect of the OPCs in the prevention and treatment of atherosclerosis. Oxidation of low-density lipoproteins (OxLDL) has been strongly suggested as a key factor in the pathogenesis of atherosclerosis [11]. Due to the antioxidant action of the proanthocyanidins and the modulation of macrophage differentiation, Mohana et al. reported the benefit of the product as prevention and treatment of the atherosclerosis [11].

Wang et al. have also tested the anti-inflammatory mechanisms of OPCs [12]. They designed an in vivo study in mouse models to investigate the protective effect of OPCs against alcohol-induced liver steatosis and injury. The results showed that OPC significantly improved alcohol-induced dyslipidemia and alleviated liver steatosis by reducing levels of serum alanine aminotransferase (ALT), aspartate aminotransferase (AST), total triglyceride (TG), total cholesterol (TC), low-density cholesterol (LDL-c), and liver malondialdehyde (MDA) and increasing levels of serum high-density lipoprotein (HDL-c) and liver superoxide dismutase (SOD). Further investigation indicated that OPC markedly decreased the expressions of lipid synthesis genes and inflammation genes such as sterol regulatory element binding protein-1c (Srebp-1c), protein-2 (Srebp2), interleukins IL-1b and IL-6, and TNF-*α* 10.

These studies suggested that an effect in the inflammation genes and mediators could be modified by the OPCs, reducing the inflammation obtained [11, 12]. Our results do not show significant differences in the IL-6 between the baseline and the second visit and between the baseline and the third visit. Significant differences were found between the 14th and 21st days and between groups in each visit, including in the baseline stage, so the groups were not similar at the beginning of the study, which could be a bias in the results. That reason forces us to not consider significantly the results obtained. However, the data is lower in the experimental group in each visit and this fact suggests that an effect could be in the IL-6, but we cannot assure this like in other studies because of our difference in the baseline group. However, the clinical parameters, the Löe and Silness index and gingival bleeding index, showed us that the clinical inflammation was higher in the control group than in the experimental group, finding statistically significant differences.

During the study, we observed that the deposition of plaque, Löe and Silness index, gingival bleeding index, and the brightness of the gingiva, increased sequentially, so the inflammatory factors must have also increased due to the results obtained in each parameter, although there were no differences between the study groups in the brightness of the gingiva. We attribute this fact to the mechanical irritant that the plaque constitutes itself, without taking into account its nature.

Besides the anti-inflammatory effect, OPCs have shown antimicrobial effect, reported by further studies [10–16, 23, 25]. This property also justifies testing the effectiveness of the OPCs in periodontal disease. After performing the current study, we can affirm that the OPCs have an effect in the health of the periodontal tissues, as we could see through the Silness and Löe and gingival bleeding indexes, which were significantly lower in the experimental group than in the control group, although it was not corroborated by the Turesky plaque index, where the presence of plaque was paradoxically higher by 33% in the study group than in the control group (Table 1).

These results lead us to suspect that this supplement certainly has antimicrobial effect, since the gingival bleeding index and Silness and Löe index were lower even in the presence of more dental plaque, so the nature of the plaque must be modified, possibly because of a change in the pH or by a different bacterial composition. In March 2015, Mahesh et al. published in the Contemporary Clinical Dentistry a randomized parallel clinical trial comparing a 0.2% chlorhexidine gluconate mouthwash versus a 0.6% cranberry mouthwash on *Streptococcus mutans* in 50 subjects, obtaining similar results in the colonisation of this bacteria in the plaque [25]. This study confirms the results obtained in our clinical trial, in which we hypothesised that the plaque could be different because of the minor inflammation of the periodontal tissue.

Until this moment, only one study has been published about the use of cranberry mouthwash (a component of OPCs) in periodontal disease in humans [25]. However, in 2011, Löhr et al. published an in vitro study about the antimicrobial effect of the OPCs acting in the adhesion of *Porphyromonas gingivalis* to KB cells [23]. They postulated that the OPCs had an antiadhesive effect against *P. gingivalis* in the KB cells. The results of this study showed that OPCs exert antiadhesive effects against *P. gingivalis*, concluding that OPCs may be useful for the prevention of *P. gingivalis*-associated periodontal diseases [23]. Our study does not include bacterial analysis, but our results support it due to the fact that in the experimental group the parameters showed less inflammation and less cell response to the plaque, which could be attributed to its nature.

Due to the previous study [23], we suspect that the antiadhesion effect modified the plaque of the subjects in the experimental group, making the inflammation lower. Lörh et al. showed that polyphenols interact with protein surface (e.g., gingipains, predominantly because of its activity against Arg-gingipain) and change the functionality and reactivity. Thus, OPCs mainly interact with the initial adhesion process of the bacteria to the cell membrane. This process could be extrapolated to our study, giving us an explication of our results. IL-6 has not shown significant differences, but a real effect in the inflammation of the gingiva took place in our patients; thus, the Löe and Silness index and gingival bleeding index were lower in the experimental group.

## 5. Conclusion

The nutritional supplement made of oligomeric proanthocyanidins (cranberry and vitamin C) induces an improvement in the health of the periodontal tissues, since the Silness and Löe index and gingival bleeding index were markedly lower in the experimental group, although that supplement has not been shown to have effects on the accumulation of plaque on the tooth surface. Further studies are needed to determine the nature of the plaque.

## Figures and Tables

**Table 1:** Groups characteristics.

		Experimental (*n* = 10)	Control (*n* = 10)	*p*
Sex	Men	2	5	0.674
	Women	8	5	
Years		24.6 ± 4.27	23.5 ± 1.64	0.525
Weight (Kg)		57.04 ± 9.33	66.6 ± 16.43	0.420
Height (cm)		164.7 ± 8.02	169.3 ± 8.59	0.325
Smoker	Yes	0	0	1.000
	No	10	10	
Alcohol	Yes	1	1	1.000
	No	9	9	
Medical treatment	Yes	1 (ACO)	0	0.875
	No	9	10	

**Table 2:** Silness and Löe, gingival bleeding, and plaque index.

Test	Evaluation visits	Experimental	Control	*p*
Silness and Löe index	Day 14	0.65 ± 0.58	1.24 ± 0.66	0.0001
	Day 21	0.97 ± 0.54	1.48 ± 0.60	0.0001
Gingival bleeding index	Day 14	0.91 ± 0.84	1.33 ± 0.75	0.005
	Day 21	0.98 ± 0.67	1.61 ± 0.66	0.0001
Plaque index	Day 14	1.68 ± 0.79	1.25 ± 0.91	0.006
	Day 21	3.35 ± 1.59	1.68 ± 1.43	0.0001

**Table 3:** Brightness of the gingiva.

Brightness of the gingiva	Experimental	Control	*p*
Baseline	30.75 ± 4.19	31.37 ± 3.55	0.351
Day 14	32.70 ± 3.48	31.50 ± 3.10	0.480
Day 21	32.57 ± 4.72	31.22 ± 2.52	0.425
Dif (day 21 and day 14)	−0.12 ± 1.85	−0.28 ± 1.92	0.545

**Table 4:** IL-6 in the crevicular fluid (pg/ml).

IL-6 in the crevicular fluid (pg/ml)	Experimental	Control	*p*
Baseline	62.85 ± 45.8	126.60 ± 54.03	0.011
Day 14	17.95 ± 13.02	47.40 ± 33.61	0.019
Day 21	22.15 ± 15.14	69.40 ± 50.10	0.011
Dif (day 14—baseline)	44.90 ± 44.05	79.20 ± 64.54	0.095
Dif (day 21—baseline)	−40.70 ± 45.02	−56.70 ± 70.63	0.245
Dif (day 21 and day 14)	−4.20 ± 3.21	−22.00 ± 21.03	0.016
